# Mining the *LIPG* Allelic Spectrum Reveals the Contribution of Rare and Common Regulatory Variants to HDL Cholesterol

**DOI:** 10.1371/journal.pgen.1002393

**Published:** 2011-12-08

**Authors:** Sumeet A. Khetarpal, Andrew C. Edmondson, Avanthi Raghavan, Hemanth Neeli, Weijun Jin, Karen O. Badellino, Serkalem Demissie, Alisa K. Manning, Stephanie L. DerOhannessian, Megan L. Wolfe, L. Adrienne Cupples, Mingyao Li, Sekar Kathiresan, Daniel J. Rader

**Affiliations:** 1 Institute for Translational Medicine and Therapeutics, Institute for Diabetes, Obesity, and Metabolism, and Cardiovascular Institute, University of Pennsylvania School of Medicine, Philadelphia, Pennsylvania, United States of America; 2 Section of Hospital Medicine, Temple University Hospital, Philadelphia, Pennsylvania, United States of America; 3 Department of Cell Biology, State University of New York Downstate Medical Center, Brooklyn, New York, United States of America; 4 University of Pennsylvania School of Nursing, Philadelphia, Pennsylvania, United States of America; 5 Department of Biostatistics, Boston University School of Public Health, Boston, Massachusetts, United States of America; 6 Framingham Heart Study, National Heart, Lung, and Blood Institute, Framingham, Massachusetts, United States of America; 7 Department of Biostatistics and Epidemiology, University of Pennsylvania School of Medicine, Philadelphia, Pennsylvania, United States of America; 8 Cardiovascular Research Center and Center for Human Genetic Research, Massachusetts General Hospital and Harvard Medical School, Boston, Massachusetts, United States of America; 9 Broad Institute of MIT and Harvard, Cambridge, Massachusetts, United States of America; Georgia Institute of Technology, United States of America

## Abstract

Genome-wide association studies (GWAS) have successfully identified loci associated with quantitative traits, such as blood lipids. Deep resequencing studies are being utilized to catalogue the allelic spectrum at GWAS loci. The goal of these studies is to identify causative variants and missing heritability, including heritability due to low frequency and rare alleles with large phenotypic impact. Whereas rare variant efforts have primarily focused on nonsynonymous coding variants, we hypothesized that noncoding variants in these loci are also functionally important. Using the HDL-C gene *LIPG* as an example, we explored the effect of regulatory variants identified through resequencing of subjects at HDL-C extremes on gene expression, protein levels, and phenotype. Resequencing a portion of the *LIPG* promoter and 5′ UTR in human subjects with extreme HDL-C, we identified several rare variants in individuals from both extremes. Luciferase reporter assays were used to measure the effect of these rare variants on *LIPG* expression. Variants conferring opposing effects on gene expression were enriched in opposite extremes of the phenotypic distribution. Minor alleles of a common regulatory haplotype and noncoding GWAS SNPs were associated with reduced plasma levels of the *LIPG* gene product endothelial lipase (EL), consistent with its role in HDL-C catabolism. Additionally, we found that a common nonfunctional coding variant associated with HDL-C (rs2000813) is in linkage disequilibrium with a 5′ UTR variant (rs34474737) that decreases *LIPG* promoter activity. We attribute the gene regulatory role of rs34474737 to the observed association of the coding variant with plasma EL levels and HDL-C. Taken together, the findings show that both rare and common noncoding regulatory variants are important contributors to the allelic spectrum in complex trait loci.

## Author Summary

Genetic association studies have identified genomic regions that affect quantifiable traits such as lipid levels. When a gene and a trait are found to be associated with one another, the gene is often further studied to determine its role in affecting the trait. One approach is to sequence the gene in individuals at the extremes of the trait's distribution with the hope of finding rare mutations that directly contribute to the trait. Until now studies using this approach have focused on genetic variation in the protein coding sequence of these genes and have been largely successful in identifying functionally important mutations. However, other studies have found an abundance of noncoding variation in the genome that may also contribute to the heritability of these traits. Here we seek to determine the contribution of such noncoding mutations to high density lipoprotein cholesterol (HDL-C) levels in humans using the HDL-C candidate gene *LIPG* as an example. Through a sequencing study in individuals with high and low HDL-C levels, we demonstrate that both rare and common noncoding mutations are influential contributors to the allelic spectrum of such traits and should be further characterized after initial association with the trait.

## Introduction

Numerous studies have associated low levels of high density lipoprotein cholesterol (HDL-C) with an increased risk of developing coronary heart disease (CHD) [1], [2], [3], [4], [5], [6], [7]. HDL-C levels are approximately 50% heritable [8]. Genome-wide association studies (GWAS) for lipid traits have identified many genes previously associated with HDL metabolism and numerous novel loci [9], [10], [11], [12], [13], [14]. However, the identification of the causal variants in these loci has proven difficult. Resequencing studies have not identified common coding variants that explain the associations. Such results may suggest that causal coding variants are rarer than anticipated [15] or lie in the gene regulatory regions. Furthermore, many of the variants identified by GWAS are embedded in gene deserts. Although a portion of these associated variants may tag less-common variants with strong phenotypic effects, some noncoding variants are likely to be causal themselves [16]. Nevertheless, combining the variation explained by all of the common variants identified to date leaves missing heritability [17] that may be explained, at least in part, by rare variants.

Several HDL-C candidate genes, including those with known physiological relevance to HDL-C metabolism, have been characterized though targeted gene-resequencing approaches [18]. Through these studies, the exons of HDL-C candidate genes (*ABCAI*, *APOAI*, *LCAT*) [19] and other mechanistically implicated genes (*ANGPTL4*, *LIPG*) [20], [21] have been sequenced in individuals at the extremes of the HDL-C phenotypic distribution. Rare coding loss-of-function variants were shown to segregate with the phenotype in a manner consistent with the known physiological role of the gene product in increasing or decreasing HDL-C levels. Causality of the identified variants was shown through a combination of *in vitro* functional studies and computational methods. Because the occurrence of each rare variant was too low to test its association in our sequencing cohorts, individual variants in each phenotypic extreme were grouped together (“collapsed”), and the total number of rare variants in the sequenced region was compared between cohorts. This method of rare variant association analysis, known as the cohort allelic sums test (CAST) [22], [23], has been instrumental in showing that rare loss-of-function variants modulate HDL-C levels in humans. However, few studies to date have utilized this approach to study rare regulatory variants, which do not always segregate with the phenotypic extremes of continuous traits as stringently as deleterious nonsynonymous variants. Additionally, the functional validation of identified variants in regulatory regions can be challenging, especially for unknown promoter or regulatory elements.

In the last decade, several HDL-C candidate genes have been identified, including many with large regulatory regions implicated in association studies. These findings, combined with the fact that HDL-C exists as a continuously distributed trait, make HDL-C candidate genes well-suited for understanding how rare regulatory variants influence complex traits. One HDL-C candidate gene associated in GWAS is *LIPG* [9], [10], [11], [12], [13], [24], [25], which encodes endothelial lipase (EL), a conserved plasma phospholipase expressed from endothelial cells [26], [27]. Compared to other plasma proteins, EL exhibits preferential HDL phospholipolysis activity *in vitro* [28]. Somatic overexpression of EL in mice causes a dose-dependent reduction in plasma HDL-C levels [29], whereas targeted deletion of *LIPG* [30] or inhibition of EL using polyclonal antibodies [31] raises HDL-C levels *in vivo*.

We recently identified rare loss-of-function coding variants in subjects with high HDL-C through a resequencing study of subjects at the extremes of the HDL-C phenotypic distribution [20]. Here, we expand our initial resequencing effort to include regulatory variations, thereby further characterizing the allelic spectrum of *LIPG*. Our findings show that both rare and common variations in regulatory regions of *LIPG* affect *LIPG* expression, plasma EL protein concentrations, and HDL-C levels.

## Results

### Identification and functional assessment of novel rare LIPG regulatory variants

**Figure 1:**
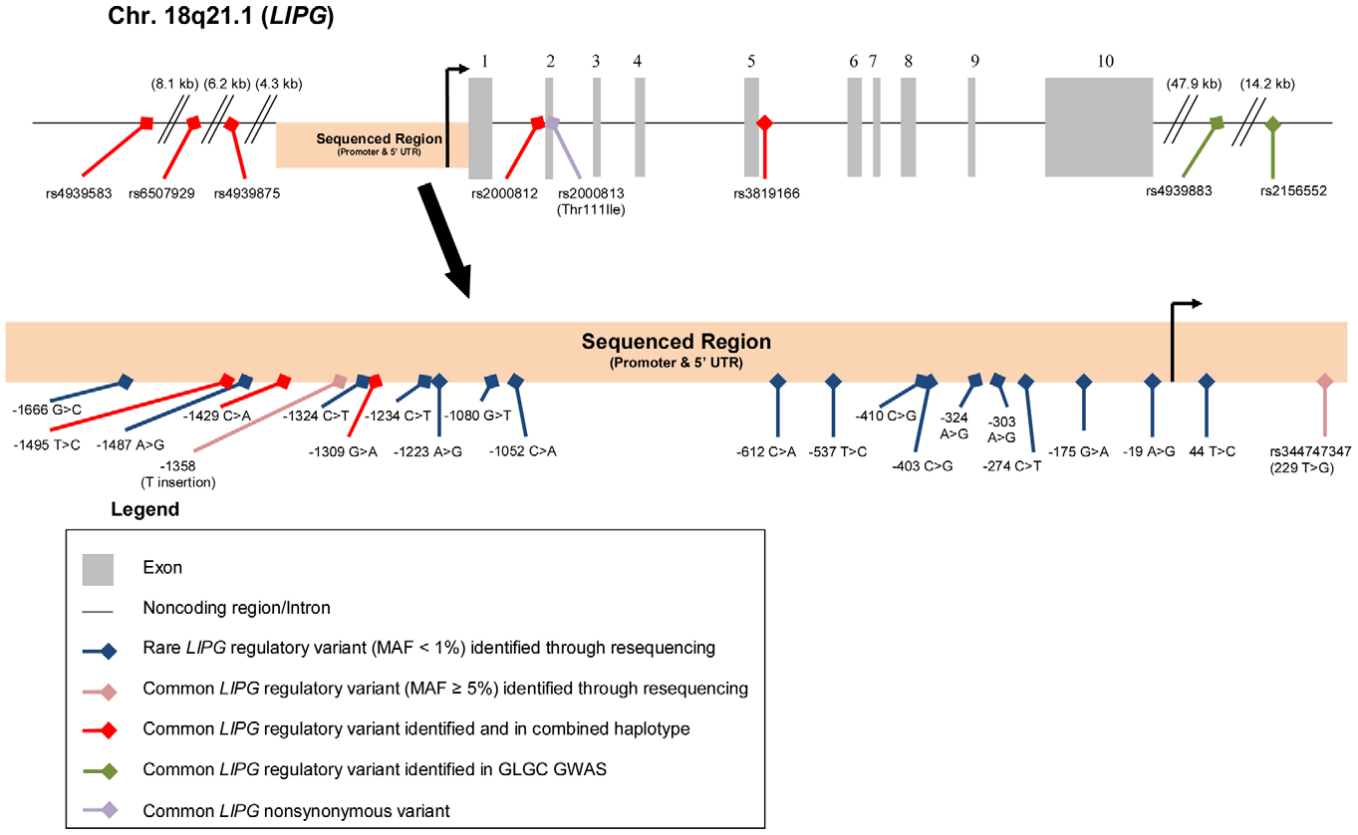
Rare and common *LIPG* regulatory variants studied. Diagram of Chr. 18q21.1 region containing *LIPG* with variants identified annotated.

We sequenced a portion of the promoter and the 5′ UTR (1755-bp immediately upstream of the transcription start site) in 388 unrelated individuals. Of the sequenced individuals, 195 individuals had extremely high HDL-C levels (≥95^th^ percentile; HHDL Sequencing Cohort) and 193 had low HDL-C levels (≤25^th^ percentile; LHDL Sequencing Cohort). A summary of the characteristics of the participants in the sequencing cohorts appears in Table 1. Through this study, we identified a total of 22 rare and common *LIPG* regulatory variants in the region sequenced (Figure 1).

**Table 1:** Baseline characteristics of participants in *LIPG* promoter resequencing.

	HHDL Sequencing Cohort (N = 195)	LHDL Sequencing Cohort (N = 193)
Ascertainment	Physician referral, HDL≥95th PCTL	Physician referral, HDL≤25th PCTL
Ethnic composition	92.2% Caucasian, 7.8% Black	91.7% Caucasian, 8.3% Black
Mean age (y)	60.4±11.9	57.5±13.9
Female (%)	58	58
HDL (mg/dL)	109.1±17.9	33.9±6.2
BMI (kg/m^2^)	23.2±2.8	29.3±5.5

Values are given as mean ± standard deviation except for ethnic composition and sex; PCTL, percentile.

25 individuals from our sequencing cohorts harbored a rare variant (minor allele frequency [MAF]<1%) in the proximal promoter or 5′ UTR of *LIPG*. Of these 25 individuals, 16 were in the HHDL and 9 were in the LHDL Sequencing Cohort (Table 2). The main characteristics of each of these participants are summarized in Table S1. Of the 17 individual rare *LIPG* regulatory mutations we identified, 10 were found only in individuals with high HDL-C, 5 occurred only in individuals with low HDL-C, and the remaining 2 occurred in individuals from both cohorts. We did not find a disproportionate frequency of rare regulatory variants between the HHDL and LHDL cohorts (*P* = 0.2142, Table 3).

**Table 2:** Identified rare^a^ *LIPG* regulatory variants.

Mutation^b^	High HDL^c^	Low HDL^d^	HDL-C (mg/dL)
***Variants identified in HHDL Sequencing Cohort***			
−1487 A>G	2	0	124–132
−1324 C>T	1	0	104
−1234 C>T	1	0	102
−1080 G>T	3	0	85–132
−612 C>A	1	0	152
−537 T>C	1	0	100
−410 C>G	1	0	110–114
−403 C>G	2	0	102–108
−274 C>T	2	0	106–108
−19 A>G	1	0	90
***Variants identified in LHDL Sequencing Cohort***			
−1666 G>C	0	2	34–36
−1223 A>G	0	1	33
−1052 C>A	0	1	34
−175 G>A	0	1	37
44 T>C	0	1	38
***Variants identified in both sequencing cohorts***			
−324 A>G	3	1	82–102 (HHDL), 44 (LHDL)
−303 A>G	1	2	124 (HHDL), 28–36 (LHDL)

a Rare *LIPG* promoter variants were defined as those with a minor allele frequency (MAF) of <0.01 as determined by the number of participants with each variant relative to the total.

b Relative to transcription start site.

c Number of individuals with the mutation identified in HHDL Sequencing Cohort.

d Number of individuals with the mutation identified in LHDL Sequencing Cohort.

**Table 3:** Association of rare *LIPG* regulatory variants with HDL-C phenotypic extremes.

Discovery cohort	Variants identified	Individualswith variant^b^	Association with discovery cohort (P value)^c^	Functional variants (effect direction)^d^	Individuals with variant decreasing promoter activity	Association for variants decreasing promoter activity (P value)^e^	Individuals with variant increasing promoter activity	Association for variants increasing promoter activity (P value)^f^	Individuals with exclusive variant decreasing promoter activity^g^	Association for exclusive variants decreasing promoter activity (P value)^h^	Individuals with exclusive variant increasing promoter activity^g^	Association for exclusive variants increasing promoter activity^i^
***HHDL Sequencing Cohort***	−1487 A>G	16	0.2142	−1487 A>G (↓)	6	0.0301	1	0.0364	6	0.0301	0	0.0297
	−1324 C>T			−1080 G>T (↓)								
	−1234 C>T			−537 T>C (↓)								
	−1080 G>T			−410 C>G (↓)								
	−612 C>A											
	−537 T>C			−303 A>G (↑)^a^								
	−410 C>G											
	−403 C>G											
	−274 C>T											
	−19 A>G											
	−324 A>G^a^											
	−303 A>G^a^											
***LHDL Sequencing Cohort***	−1666 G>C	9		−1666 G>C (↑)	0		7		0		5	
	−1223 A>G			−1223 A>G (↑)								
	−1052 C>A			−1052 C>A (↑)								
	−175 G>A			−175 G>A (↑)								
	44 T>C											
	−324 A>G^a^			−303 A>G (↑)^a^								
	−303 A>G^a^											

a Rare variants found in individuals from both HHDL and LHDL Sequencing Cohorts.

b Individuals were included if they harbored at least 1 rare *LIPG* regulatory variant of those identified. Three individuals from the HHDL Sequencing Cohort had two rare regulatory mutations each: one with −1487 A>G and −1080 G>T, one with −1234 C>T and −324 A>G, and one with −1487 A>G and −303 A>G. All three individuals were included once each in the total counts.

c The number of individuals with a rare variant was compared between the 2 cohorts with a 2-tailed Fisher's exact test. All rare variants were considered, regardless of functional impact on *LIPG* expression and including variants found in both sequencing cohorts.

d Functional variants were found to alter *LIPG* promoter activity relative to WT *in vitro* by luciferase reporter assays (Figure 2).

e Variants decreasing promoter activity were tested for association with the HHDL Sequencing Cohort with a 2-tailed Fisher's exact test. All functional variants decreasing promoter activity were tested, including variants found in both sequencing cohorts.

f Variants increasing promoter activity were tested for association with the LHDL Sequencing Cohort with a 2-tailed Fisher's exact test. All functional variants increasing promoter activity were tested, including variants found in both sequencing cohorts.

g Exclusive variants are defined as variants occurring in individuals in either of the 2 sequencing cohorts.

h Number of individuals with a rare exclusive variant decreasing promoter activity was compared between the 2 cohorts via 2-tailed Fisher's exact test.

i Number of individuals with a rare exclusive variant increasing promoter activity was compared between the 2 cohorts via 2-tailed Fisher's exact test.

We also searched for these variants in the 1000 Genomes Project database (451 participants; [32]) and found that only the 2 variants present in both cohorts, −303 A>G and −324 A>G, occurred in individuals of the YRI ethnicity in this database (MAF = 0.014 for −303 A>G, MAF = 0.024 for −324 A>G). Neither of these variants was present in 1000 Genomes Project participants of other ethnicities, nor were any of the other 15 variants present in any population from this study.

To determine the functional significance of the identified variants in modulating *LIPG* promoter activity, variants were tested with a luciferase reporter assay in HUVECs, which endogenously express *LIPG*. A wild-type *LIPG* promoter construct corresponding to the sequenced portion of the *LIPG* promoter was constructed and tested against the promoter-less pGL3-basic construct. The WT *LIPG* promoter construct displayed approximately 31.9 times greater relative luciferase activity than the pGL3-basic construct (Figure S1).

We tested promoter constructs corresponding to the rare *LIPG* variants. Four of the 10 rare variants found only in high HDL-C individuals displayed decreased promoter activity relative to the WT promoter construct (Figure 2A). In contrast, 4 of the 5 rare variants found only in low HDL-C individuals displayed increased promoter activity (Figure 2B). The remaining 6 variants identified in only in high HDL-C individuals and 1 variant identified only in low HDL-C individuals did not alter promoter activity relative to WT (Figure S2A and S2B). One of the 2 rare regulatory variants found at both extremes (−303 A>G) caused increased promoter activity *in vitro* (Figure S2C). Six individuals from the HHDL Sequencing Cohort had a rare regulatory variant decreasing *LIPG* expression in vitro, compared to no individuals from the LHDL Sequencing Cohort (*P* = 0.0301, Fisher's exact test, Table 3). One individual from the HHDL Sequencing Cohort had a rare regulatory variant increasing promoter activity, compared with 7 individuals from the LHDL Sequencing Cohort (*P* = 0.0364, Table 3).

**Figure 2:**
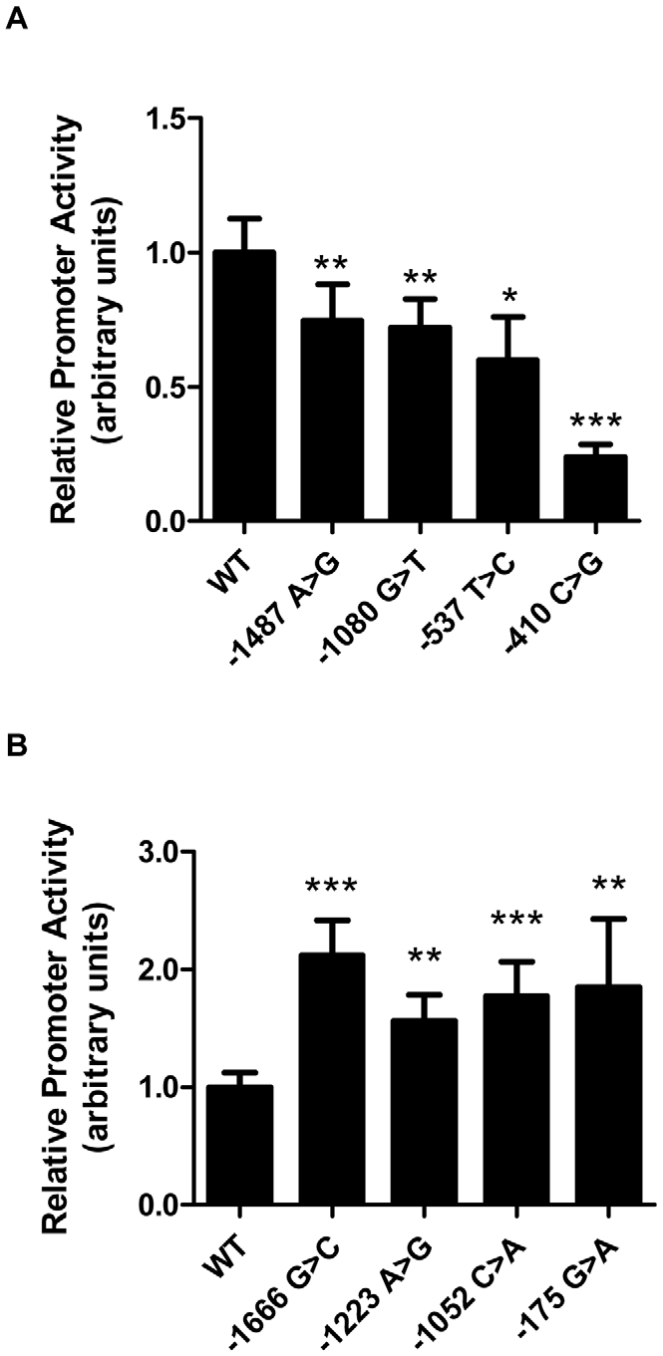
Rare *LIPG* regulatory variants modulate transcriptional activity *in vitro*. Relative promoter activity of rare variants (MAF<0.01) identified from resequencing of high HDL-C individuals (A) or low HDL-C individuals (B). Plasmid constructs expressing firefly luciferase under the control of wild-type (WT) or variant *LIPG* promoters were individually co-transfected with a Renilla luciferase reporter construct (pRL-SV40) in HUVECs. Firefly luciferase expression were measured and normalized to that of Renilla luciferase, and Renilla-normalized promoter activities for variant constructs were then compared to those of the WT construct to provide relative *LIPG* promoter activities of the variants. Assays were conducted with 6 replicates per experiment and data is given as mean ± standard deviation. *P-value<0.05, **P-value<0.01, ***P-value<0.0001, compared with WT.

Next, we individually compared the number of individuals with functional rare regulatory variants identified in either sequencing cohort. We excluded the 2 regulatory mutations that were identified in individuals from both cohorts and reassessed the association of functional rare regulatory variants with the phenotypic extremes. Similar to the results obtained above, a significant excess of rare *LIPG* promoter variants causing decreased *LIPG* expression was found in individuals with high HDL-C (*P* = 0.0301, Table 3), and an excess of rare variants causing increased promoter activity was found in individuals with low HDL-C (*P* = 0.0297, Table 3). Notably, when we enriched for variants only present in either of the cohorts, no variants decreasing *LIPG* promoter activity *in vitro* were identified in individuals with low HDL-C. Likewise, no variants increasing promoter activity were present in individuals with high HDL-C.

### Identification and association of common LIPG regulatory variants associated with HDL-C

In addition to discovering novel, rare *LIPG* regulatory variants, our sequencing effort identified 5 common variants (MAF≥5%), all of which were present in both high HDL-C and low HDL-C subjects (Figure 1 and Table 4). The minor alleles of 3 of the identified variants (rs9959847, −1495 T>C; rs4245232, −1429 C>A; rs3829632, −1309 A>G) are in complete LD with each other and constitute a common haplotype. According to the International HapMap Project dataset [33], this haplotype includes 3 additional SNPs upstream of the sequenced region (rs4939583, rs6507929, rs4939875) and 2 intronic SNPs (rs2000812, rs3819166) (Figure 1, Table S2, Figure S3). We assessed the association of 2 of the identified common variants, −1309 A>G (rs3829632) and −1358 (T insertion), with HDL-C and other HDL traits in the Framingham Heart Study Offspring cohort (FHS; 1089 subjects in this analysis, Table 5). The −1309 A>G variant was used as a tag SNP for the haplotype. Although the −1358 (T insertion) variant had a borderline association with decreased HDL_3_ subfraction, the −1309 A>G variant (and, thus, the entire haplotype) was strongly associated with decreased HDL-C by approximately 2 mg/dL (*P*<0.0002). This latter variant was also associated with decreases in HDL_3_, large HDL particles, apoA-I (the major protein component of all HDL), and HDL size. Consistent with these findings, a recent GWAS of >100,000 individuals by the Global Lipids Genetics Consortium (GLGC) found that the minor alleles of several variants of this haplotype were strongly associated with a reduction in HDL-C (Table S2) [13]. Neither the −1358 (T insertion) or −1309 A>G variants were associated with changes in any other lipid or lipoprotein measures in the FHS (data not shown).

**Table 4:** Identified common^a^ *LIPG* regulatory variants.

Mutation^b^	Genotype^c^	High HDL^d^	Low HDL^e^
−1495 T>C (rs9958947)^f^	Homozygous	5 (2.6%)	9 (4.7%)
	Heterozygous	44 (22.6%)	55 (28.5%)
	MAF^h^	0.14	0.19
−1429 C>A (rs4245232)^f^	Homozygous	5 (2.6%)	9 (4.7%)
	Heterozygous	44 (22.6%)	55 (28.5%)
	MAF	0.14	0.19
−1309 A>G (rs3829632)^f^	Homozygous	5 (2.6%)	9 (4.7%)
	Heterozygous	44 (22.6%)	55 (28.5%)
	MAF	0.14	0.19
−1358 T insertion^g^	Homozygous	1 (0.5%)	0 (0%)
	Heterozygous	17 (8.7%)	23 (11.9%)
	MAF	0.05	0.06
229 T>G (rs34474737)	Homozygous	25 (12.8%)	16 (8.4%)
	Heterozygous	96 (49.2%)	103 (55.1%)
	MAF	0.37	0.35

a Common *LIPG* promoter variants were defined as those with MAF≥0.05 as determined by number of participants with each variant relative to the total.

b Relative to transcription start site.

c Homozygous and heterozygous refer to minor allele.

d Number identified in HHDL Sequencing Cohort (percentage of total sequenced in cohort).

e Number identified in LHDL Sequencing Cohort (percentage of total sequenced in cohort).

f Minor alleles of −1495, −1429, and −1309 variants were present in a common haplotype.

g A rs number for this SNP was not present in dbSNP.

h Minor allele frequency, as determined for each cohort.

**Table 5:** Association of common variants with HDL in Framingham Heart Study.

*Variant*	−1358 (T insertion)		−1309 A>G (rs3829632)		rs4939883		rs2156552	
Phenotype	Δ S.D.^a^	P value	Δ S.D.^a^	P value	Δ S.D.^a^	P value	Δ S.D.^a^	P value
**HDL**	−0.09	0.15	−0.15	0.0002	−0.16	2.28×10^−7^	−0.18	1.08×10^−8^
**HDL_2_**	−0.01	0.84	−0.07	0.14	−0.10	0.002	−0.16	5.14×10^−7^
**HDL_3_**	−0.17	0.02	−0.12	0.01	−0.15	2.59×10^−5^	−0.13	0.0002
**HDL size**	−0.07	0.36	−0.12	0.01	−0.11	0.004	−0.12	0.002
**HDL small particle**	−0.07	0.38	0.01	0.88	0.06	0.08	0.06	0.13
**HDL intermediate particle**	0.04	0.62	0.07	0.16	−0.07	0.04	−0.07	0.05
**HDL large particle**	−0.1	0.22	−0.14	0.004	−0.14	0.0002	−0.15	9.31×10^−5^
**apoA-I**	−0.11	0.13	−0.09	0.05	−0.12	0.0003	−0.13	0.0001

a ΔSD represents the fractional change in standard deviation (SD) in standardized residual (mean = 0, SD = 1 after adjustment for age, age^2^, BMI, alcohol intake, smoking status, menopause, and hormone replacement therapy separately by gender) per copy of minor allele. One SD unit in the Framingham Heart Study was 13.2 mg/dL.

### Functional analysis of common LIPG regulatory variants

**Figure 3:**
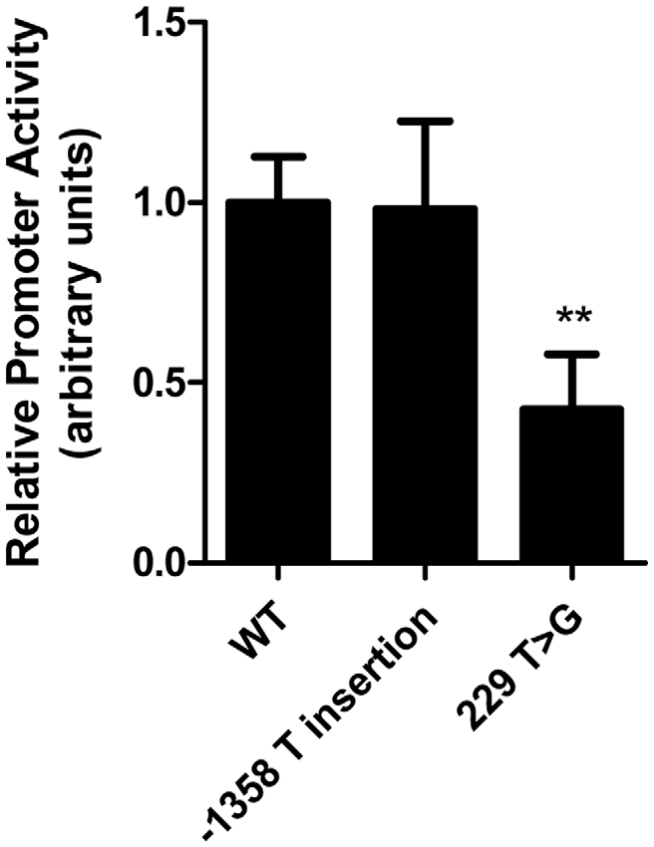
Common *LIPG* regulatory variant rs34474737 affects *LIPG* promoter activity *in vitro*. Relative *LIPG* promoter activity of common variants rs34474737 (229 T>G) and −1358 T insertion variant identified from resequencing of individuals with high and low HDL-C levels, measured as relative firefly luciferase expression of *LIPG* variant constructs in HUVECs. Assays were conducted with 6 replicates per experiment and data is given as mean ± standard deviation. **P-value<0.01, compared with WT.

Reporter constructs corresponding to the common *LIPG* regulatory variant rs34474737 (229 T>G) and the −1358 T insertion variant, neither of which is known to be part of a haplotype extending beyond the *LIPG* promoter, were generated and used to test their impact on *LIPG* promoter activity in HUVECs (Figure 3). The rs34474737 variant caused a marked reduction in luciferase reporter activity (*P*<0.01 vs. WT), whereas the −1358 (T insertion) variant, which was not strongly associated with modulation of HDL-C in FHS, did not significantly alter *LIPG* promoter activity.

We hypothesized that the common *LIPG* regulatory variant rs34474737, which decreases promoter activity *in vitro*, would cause decreased plasma levels of EL in human subjects. If true, this finding would provide a mechanism through which the identified variants could increase HDL-C levels in humans. We also assessed the role of 2 recently associated noncoding variants (rs2156552 and rs4299883) and the common haplotype spanning the *LIPG* locus (rs3829632, −1309 A>G) in the regulation of *LIPG* expression, by testing the effects of these variants on plasma EL. The EL concentrations were measured in participants of the SIRCA study who were genotyped for variants rs34474737 (n = 761), rs2156552 (n = 570), rs4299883 (n = 755), and rs3829632 (n = 760) (Table 6).

**Table 6:** Association of common *LIPG* variants with plasma EL concentrations in SIRCA.

*LIPG* Variant (ref. allele>minor allele)	MAF	Effect of minor allele on HDL-C (P value)^e^	Genotype (N^f^)	Mean plasma EL^g^	Effect of minor allele on plasma EL (P value)^h^
Combined Haplotype^a^	0.221^a^	↓ (8.64×10^−10^)^a^	AA (537)^a^	483.5±342.1^a^	↑ (0.041)^a^
			AG (202)	538.4±368.9	
			GG (21)	540.2±367.9	
rs4939883^b^ (C>T)	0.190^c^	↓ (4.34×10^−49^)	CC (542)	481.8±353.5	↑ (1.43×10^−3^)
			CT (203)	538.8±342.0	
			TT (10)	772.9±452.8	
rs2156552^b^ (T>A)	0.190^c^	↓ (5.53×10^−45^)	TT (419)	486.2±362.1	↑ (3.48×10^−3^)
			TA (146)	545.1±346.5	
			AA (5)	852.1±565.7	
rs34474737 (229 T>G)	0.278^d^	N/A	TT (391)	520.1±338.3	↓ (3.38×10^−3^)
			TG (305)	505.7±379.3	
			GG (70)	417.6±302.0	
rs2000813 (584 C>T; Thr111Ile)	0.279^c^	↑ (1.92×10^−14^)	CC (352)	521.2±343.5	↓ (7.00×10^−4^)
			CT (330)	492.7±362.9	
			TT (79)	417.4±319.9	

a Haplotype block containing variants rs3829632, rs4245232, rs9958947, rs4939875, rs6507929, rs4939583, rs3819166, and rs2000812. Representative minor allele frequency (MAF; from HapMap, CEU Set, Caucasians from Utah, USA) and effect of minor allele on HDL-C in GLGC GWAS are given for the rs4245232 variant. Genotyping in SIRCA was completed for the rs3829632 variant and this was used to assess association of the minor allele of this variant with mean plasma EL concentration. Individual variants, their chromosomal location, and P values for the association of the minor alleles with HDL-C in the Global Lipids Genetics Consortium (GLGC) GWAS are given in Table S2. R^2^ values for LD of individual variants of the haplotype block are given.

b Identified in the GLGC GWAS [13].

c From HapMap (CEU Set, Caucasians from Utah, USA).

d From dbSNP (CEU Set, Caucasians from Utah, USA).

e Association with HDL-C in the GLGC GWAS.

f Number of individuals in SIRCA with given genotype whose plasma EL concentrations were measured.

g Pre-heparin plasma EL concentrations (ng/mL), shown as mean ± S.D.

h Association of log-transformed mean plasma EL concentration with minor allele for each variant in SIRCA.

Minor alleles of the rs4299883 and rs2156552 variants were highly associated with decreased HDL-C in the GLGC GWAS (P<10^−44^ and P<10^−48^ respectively) [13]. We tested the association of these 2 variants with HDL-C and HDL subphenotypes in the FHS, and found that the minor alleles of these variants are associated with decreased HDL-C, HDL_2_, HDL_3_, and HDL particle sizes and apoA-I levels (Table 5). Consistent with these findings, the minor alleles of these variants were also associated with increased plasma EL (*P*<0.002 and P<0.004, respectively) (Table 6). The minor allele of the −1309 A>G variant was moderately associated with increased plasma EL (*P*<0.05), consistent with its role in decreasing plasma HDL-C, as suggested by the GLGC and FHS association studies.

The minor allele of the rs344747347 (229 T>G) variant was highly associated with decreased plasma EL (*P*<0.004), consistent with the luciferase reporter assay results. Plasma EL concentrations were measured for individuals in SIRCA genotyped for the rs2000813 variant (Thr111Ile; n = 761). This common nonsynonymous variant does not alter EL lipolytic activity *in vitro* or *in vivo* [20], but was associated with increased HDL-C in GLGC (*P* = 1.92×10^−14^). Plasma EL concentrations decreased with the minor allele of the Thr111Ile variant (P<0.0008, Table 6).

**Figure 4:**
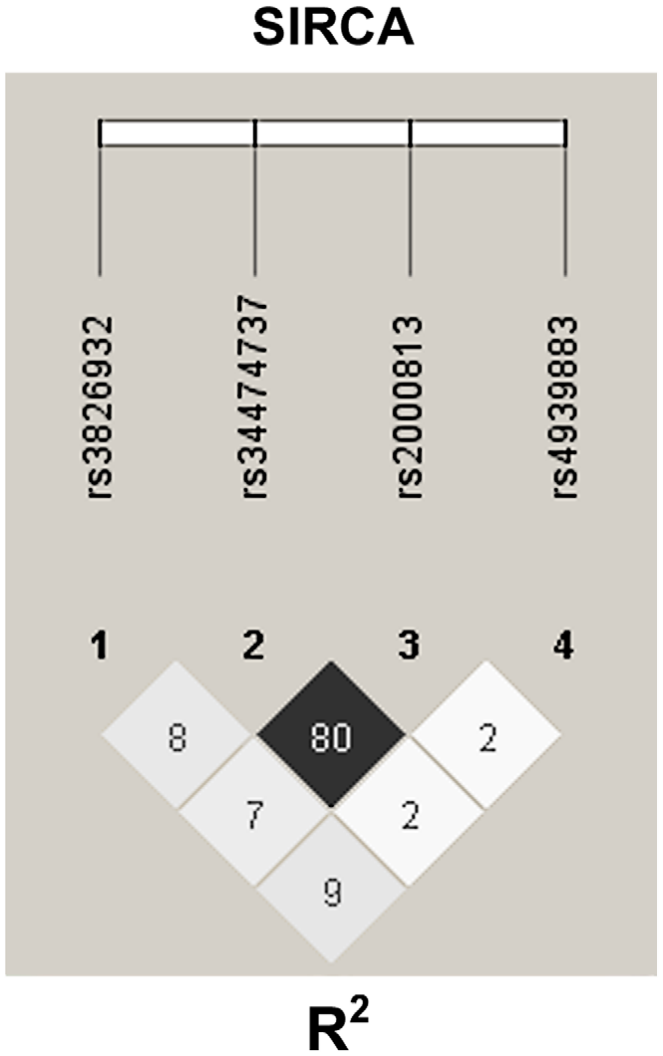
Linkage disequilibrium of rs34474737 (229 T>G) and rs2000813 (Thr111Ile) variants. Genotyping of rs34474737 and rs2000813 variants was completed in SIRCA participants (761 in total). LD was estimated and plotted using this genotyping data using Haploview software. Values in the LD plot are estimated squared correlation coefficients (R^2^).

It may be that the Thr111Ile variant is in high LD with a regulatory variant that decreases EL expression, which would explain the decreased plasma EL of subjects with the Thr111Ile variant, as well as its association with HDL-C but normal lipolytic activity in GLGC. To test this possibility, using genotyping data for the common regulatory variants in SIRCA participants, we estimated their LD with Haploview software [34]. The rs34474737 (229 T>G) and rs2000813 (Thr111Ile) variants were in high LD (R^2^ = 0.8) (Figure 4).

## Discussion

GWAS and candidate gene association techniques clearly contribute to the identification and validation of candidate genes for complex traits; however, they have fallen short in identifying causal variations. Although rare variants hold much promise for filling this void [35], the association of rare mutations with continuously distributed phenotypes has been hampered by the dual presence of functional and nonfunctional mutations. Moreover, studies have shown a lack of uniformity in incorporating the functional relevance of rare variants into their analyses. The direct influence of regulatory variants, for which functional significance is often ambiguous, also remains largely uncharacterized.

To address the phenotypic contributions of rare and common regulatory variants, we utilized the continuous trait HDL-C and candidate gene *LIPG*, which has significant genome-wide common and causal coding variations. By uncovering the allelic spectrum of *LIPG* regulatory regions through sequencing at the HDL-C extremes, rare and common regulatory mutations in *LIPG* were shown to contribute to observable variation in HDL-C levels. The findings also demonstrated that the functional impact of identified variants can help guide statistical analyses that assess their combined effect on a studied phenotype. To our knowledge, this study is one of the first applications of a rare variant association test to regulatory variants for a complex trait, as well as the first of such analyses to be informed by functional assays.

### Association tests for rare variants of complex traits

Numerous methodologies have been described for statistically comparing the frequency differences of rare coding variants for a complex trait in cases and controls [22]. Some approaches assume that much of the heritability of complex traits arises from the combined presence of functionally important rare variants. These, which include CAST and combined multivariate and collapsing (CMC) method, collapse rare variants within a functional location (e.g., gene locus) and compare the frequencies of the aggregate variants between cases and controls [36], [37]. Other methods for evaluating rare, risk-conferring mutations include weighted sum methods that count both rare and common coding variants. These tests weight variants based on their frequency in controls [38] or are informed by computational prediction programs for assessing functionality [39]. Although these assessment methods demonstrate high statistical power, they are disadvantaged by their inclusion of both rare and common variants, as well as functional information that is largely inapplicable or unavailable for noncoding variants.

Although the effects of rare missense variants are frequently deleterious with regard to protein structure and function, the effects of rare regulatory variants are less readily interpretable [23], [36]. Such variants may cause increased or decreased gene expression, depending on their location; may act in a tissue-dependent manner, thereby weakening their association with complex traits; and may increase, decrease, or not affect transcription at all. Whereas nonfunctional coding variants can be predicted easily by synonymous or conservative amino acid substitutions, similar criteria cannot be applied to regulatory variants.

We first used CAST to investigate the contribution of rare regulatory variants to HDL-C without computationally predicting their effects. The results showed no significant excess of rare regulatory variants in *LIPG* in either cohort. However, the strength of rare variant aggregation methods increases when the functional validity of the variants is known [22], [23], [40], [41]. Therefore, we assessed the functional effects of each variant in a cell type that endogenously expresses *LIPG* (HUVECs). These putative functional effects were used to reassess the association of functional variants in the 2 cohorts. Using a modification of CAST, we separately tested the associations of variants that increase or decrease *LIPG* promoter activity. The results showed that variants segregated with the phenotypic extremes in a manner that was almost completely consistent with the contribution of the gene to the phenotype. For example, given that EL inversely affects HDL-C levels, variants that decrease EL should cause increased HDL-C and should occur at a higher frequency in high HDL-C individuals, and *vice versa*. Including functional information in the association analysis permitted the near-perfect demonstration of this distribution.

The only rare regulatory mutation inconsistent with the expected distribution was the −303 A>G variant, which increased *LIPG* promoter activity *in vitro*. This variant was found in 1 high and 2 low HDL-C individuals, which is the expected distribution, given its *in vitro* functionality. However, the high HDL-C individual with the −303 A>G variant also had another rare LIPG regulatory variant, −1487 A>G, which decreased promoter activity *in vitro*. Thus, the actual role of −303 A>G in contributing to high HDL-C levels must be considered in the context of the contribution from the additional rare variant in this individual.

Previously, Hegele *et al.* presented an elegant approach of refining association tests by using exclusively presenting coding variants [42]. In the present study, this approach was modified for application to noncoding variants. We examined the association of variant types with the phenotypic extremes after eliminating variants occurring at both extremes. The results showed that promoter-activating or -damaging rare *LIPG* variants occurred only in individuals with high or low HDL-C, respectively. Thus, our analysis method effectively enriched for functional variants with the greatest potential effect at either extreme. A limitation of this approach is that the exclusivity of any rare variant depends on the selection criteria and sizes of the cohorts. Nevertheless, even without this selectivity filter, the expected enrichment of opposing regulatory variant types occurred at the opposite phenotypic extremes.

The current literature contains additional rare variant association tests that evaluate the contribution of risk and protective rare variants to complex traits. One is a modified C-alpha score-test that measures the deviation of variance of each observed mutation from the expected variance with a binomial distribution. However, this method may not be valid for evaluating variants occurring only once in a test cohort, such as were identified in our study [43]. Another method, weighted sum test, calculates 2 one-sided statistics to quantify the association of variants in either phenotypic extreme. This test allows the incorporation of functional information of the identified variants and may be applicable to measuring the association of rare regulatory variants [44]. Yet neither of these methods is sufficiently robust to manage the large number of rare nonfunctional variants likely to be identified in resequencing studies of regulatory regions. In our study, nearly half of the rare variants identified in only one extreme failed to have any transcriptional effect. A recently reported modification of a previous methodology for studying common variants, the sequence kernel association test, may prove useful in studying the association of such rare variants without making any assumption of the functional direction or degree of effect of any individual variants [45].

### Putative haplotype involving a causal regulatory and a nonfunctional coding variant of LIPG is associated with HDL-C levels

Exploration of the *LIPG* noncoding regions revealed the contributions of common regulatory variants. For example, the 229 T>G (rs34474737) variant in the 5′ UTR was found to decrease *LIPG* promoter activity *in vitro* and to raise plasma EL in humans. This variant was in LD with the common nonsynonymous variant Thr111Ile (rs2000813). Thr111Ile is a missense variant that does not damage EL function (according to the PolyPhen prediction program) and does not alter EL lipolytic activity *in vitro* or *in vivo* [20]. The association of Thr111lle with HDL is unclear, with some studies purporting a weak association with elevated HDL-C and others showing no association [46], [47], [48], [49], [50], [51], [52]. However, a recent GLGC GWAS metaanalysis of >100,000 individuals revealed significant association of this variant with increased plasma HDL-C (*P* = 1.92×10^−14^) [13], suggesting that Thr111Ile may be in LD with a regulatory variant.

Based on the high LD between 229 T>G and Thr111Ile, as well as the association of plasma EL with minor alleles of the 229 T>G and Thr111Ile variants in SIRCA participants, we propose that the 229 T>G variant may cause the association of Thr111Ile with HDL-C by decreasing plasma EL. To our knowledge, this finding represents the first identification of a putative haplotype involving a causal regulatory variant and a functionally benign coding variant. The result also highlights the potential misattribution that can occur when nonsynonymous coding variants are considered to be highly suggestive of causal mutations, and regulatory variants are ignored.

Interestingly, there are several reports of common nonsynonymous variants causing the association of noncoding variants in high LD with a phenotype. Kanda *et al.* reported that a common missense variant in high LD with a nearby promoter SNP at chromosome 10q26 independently explains the association of the locus with susceptibility to age-related macular degeneration [53]. A common functional missense variant in the B-cell scaffold protein BANK1 was shown to be in high LD with a common intronic variant that alters splicing, and both variants were strongly associated with systemic lupus erythematosus [54]. The functional heterogeneity of linked coding and noncoding SNPs highlights the complexity of haplotype structures, as well as the need to characterize the complete (i.e., coding and noncoding) variation in candidate loci for complex traits. Indeed, resequencing studies to identify haplotypes in candidate genes for inflammation, lipid metabolism, and blood pressure regulation are susceptible to missing partial or whole haplotype blocks when only coding variation is considered [55]. Common regulatory variation in observed haplotypes for several complex traits may have profound functional significance.

In our analysis of common regulatory variation in *LIPG*, we identified another haplotype with SNPs in the proximal promoter region. Three variants, −1495 T>C (rs9958947), −1429 C>A (rs4245232), and −1309 A>G (rs3829632), identified in the promoter region were in complete LD with each other. A study of HapMap data indicated that three SNPs upstream of the sequenced region (rs4839583, rs6507929, and rs4939875) and SNPs in the second and fifth introns of *LIPG* (rs2000812 and rs3819166, respectively) are also in high LD with these three promoter SNPs [33]. Because the region encompassed by this haplotype extends far upstream and within the *LIPG* gene (approximately 34.1 kb from the most 5′ to most 3′ of the variant constituents of the haplotype), it is not possible to assess its full functional impact with a reporter driven by part of the *LIPG* promoter. Characterization of the effects of single variants of this haplotype on *LIPG* expression *in vitro* could lead to erroneous implications about their functional significance, because their aggregate (and potentially synergistic) effects on transcription would be ignored. Therefore, we evaluated the contribution of the combined haplotype by measuring its effect on HDL-C levels and plasma EL concentrations from human subjects. Minor alleles of the haplotype variants were associated with decreased HDL-C in the FHS and GLGC GWAS studies, and the minor allele of 1 variant was associated with increased plasma EL. Together, these findings implicate this haplotype in the reduction of human HDL-C.

Association of minor alleles of the −1309 A>G, rs4939883, and rs2156552 variants with decreased HDL-C (*P* = 0.0002, 2.28×10^−7^, and 1.08×10^−8^, respectively) in the FHS was supported by a similar association with decreases in the HDL subphenotypes HDL_2_ and HDL_3_. A recent GWAS of 17 nonconventional, NMR-assessed lipoprotein measures also identified association of the rs4938993 variant with apoA-I and large HDL particles under both fasting and nonfasting conditions [56]. Together, these results demonstrate the reproducibility of such measurements in association studies. Future lipid genetic association studies using nonstandard measurements may provide additional insights beyond aggregate lipoprotein measures.

Finally, we evaluated 2 SNPs, rs2156552 and rs4299883, which were recently reported in the GLGC GWAS metaanalysis to be highly associated with HDL-C. Both variants are 40–65 kb downstream of the *LIPG* gene and are in high LD with each other [20], but not with Thr111Ile or Asn396Ser. In addition to being associated with decreased HDL-C, the minor alleles of these variants are associated with increased plasma EL in humans. We did not observe any LD with any of the common variants identified in our resequencing study. Further analysis of the regulatory region harboring these SNPs may help elucidate the mechanism by which these variants contribute to increased human *LIPG* expression.

The molecular regulators of *LIPG* expression are largely unknown. Investigations of induced EL secretion from human endothelial cells upon cytokine treatment have suggested that *LIPG* is regulated in an NFκB-dependent manner [57]. Subsequent studies utilizing electrophoretic mobility shift assays, chromatin immunoprecipitation (ChIP), and cotransfection experiments of luciferase reporter constructs determined that the *LIPG* promoter contains 2 NFκB binding sites, one of which (position −1250 relative to the transcription start site) exhibited strong NFκB binding *in vitro* [58]. In addition, ChIP combined with genome tiling arrays in HepG2 liver cell lines identified *LIPG* as a potential target of the SREBP1 transcription factor, a major regulator of cellular fatty acid synthesis and metabolism [59]. None of the promoter variants identified in this study disrupt the NFκB or SREBP1 binding sites. Further characterization of regulatory variants affecting *LIPG* expression may help elucidate key regulators of *LIPG* expression.

### Conclusions

In this study, we demonstrate that regulatory variants, both common and rare, causally contribute to an associated phenotype. Given the complexities of interpreting the functionality of noncoding variants, direct experimental evaluation may be required to assess their impact accurately. By expanding on previous statistical association methods, this study provides an example of how such an evaluation may be done. As future whole-genome sequencing efforts will undoubtedly uncover myriad causal regulatory mutations for several polygenic traits, the findings in this study should encourage the development of methodologies to assess the contribution of rare noncoding variants.

## Materials and Methods

### Ethics statement

Written informed consent was obtained from all participants in the cohorts described. The UPenn Institutional Review Board (IRB) approved all study protocols.

### Research participants for the sequencing cohorts

*LIPG* regulatory variants were identified in a discovery cohort of subjects selected from the extremes of the HDL-C phenotypic distribution in the following cohorts: University of Pennsylvania (UPenn) High HDL Cholesterol Study (HHDL), UPenn Catheterization cohort (PennCATH), Study of Inherited Risk of Coronary Atherosclerosis (SIRCA), and Philadelphia Area Metabolic Syndrome Network (PAMSyN).

HHDL is a cross-sectional study of genetic factors contributing to elevated HDL-C levels. Individuals with elevated HDL-C (>90^th^ percentile for age and gender) were identified by physician referrals or through the Hospital of the UPenn clinical laboratory. PennCATH is composed of consecutive subjects undergoing coronary angiography at UPenn Health System hospitals and has been previously described [60]. SIRCA is a cross-sectional study of factors associated with coronary artery calcification in asymptomatic subjects recruited on the basis of a family history of premature coronary artery disease. Study design and initial findings have been previously published [61]. PAMSyN is a cross-sectional study of individuals with varying numbers of metabolic syndrome criteria, from none to all 5.

High HDL participants and low HDL participants were chosen from these cohorts. HHDL Sequencing Cohort participants are subjects with elevated HDL-C (≥95th percentile) for age and sex (females, range 87–174 mg/dL; males, range 85–166 mg/dL). LHDL Sequencing Cohort participants are subjects with low HDL-C (≤25th percentile), excluding individuals with HDL-C <20 mg/dL to eliminate participants with likely monogenic disorders of lipoprotein metabolism, leading to reduced HDL-C concentration (females, range 22–61 mg/dL; males, range 23–44 mg/dL). Approximately 92% of participants were Caucasian, while the remaining 8% were of African descent; 42% of the participants were males, which was representative of the overall demographics of the parent studies. In total, 195 high HDL participants and 193 low HDL participants were chosen for deep resequencing analysis of the *LIPG* promoter.

### Research participants in Framingham Heart Study association

The Framingham Heart Study (FHS) Offspring Cohort, consisting of 5124 participants who were offspring of the original cohort recruited in 1948 and the spouses of the offspring, was initiated in 1971. Participants have been examined every 4 to 8 years. The examined genotypes were from a panel of 1778 unrelated individuals who provided blood samples for DNA extraction during the sixth examination cycle (1995–1998). HDL measurements were available at up to 7 time points for each individual. The HDL mean from the available measures for each individual was used. HDL_2_, HDL_3_, HDL size, HDL subfractions, and apoA-I, measured at exam 4, were determined as described previously [62], [63], [64]. The Institutional Review Board at Boston Medical Center approved the study, and all participants gave written informed consent.

### Sequencing

A 1755-bp region of the promoter region (directly upstream of the transcription start site) of *LIPG* was amplified using a polymerase chain reaction (PCR)-based strategy. Genomic DNA was isolated from peripheral blood leukocytes using Nucleon extraction and purification protocols (Amersham). PCR reactions containing 200 ng of DNA template using Ready-to-Go PCR Beads (Amersham) were amplified in a final volume of 25 µL. The PCR program included denaturation at 95°C for 5 min, followed by 35 cycles (95°C for 1 min, 61.5°C for 30 s, and 72°C for 1 min), and extension at 72°C for 2 min. PCR products were purified with ExoSAP-IT (USB, Cleveland, OH). Purified PCR products were analyzed via Sanger sequencing on an ABI sequencer with Big Dye (Applied Biosystems) terminator chemistry. Sequences were aligned and chromatograms viewed with Sequencher Version 4.8 (Gene Codes) software. Allelic variations were verified by inspecting chromatograms. Putative variants identified in the HHDL and LHDL Sequencing Cohorts were searched for in the 1000 Genomes Project database. Rare variants were those with <1% MAF in our sequencing cohorts, and common variants were those with ≥5% MAF in our sequencing cohorts.

### Genotyping

The −1309 A>G (rs3829632) and −1358 (T insertion) variants were genotyped in participants of the FHS for association analysis with HDL-C and other HDL traits by using Taqman custom genotyping assays (Applied Biosystems). For association of common variants with plasma EL concentration in SIRCA participants, genotyping was completed by using either Taqman custom genotyping assays (for −1309 A>G [rs3829632], 229 T>G [rs34474737], and rs4299883) or the ITMAT-Broad-CARe (IBC) cardiovascular gene genotyping array [65]. DNA was diluted to 50 ng/µL, and genotyping was performed at the Center for Applied Genomics (Children's Hospital of Pennsylvania) following manufacturer specifications for amplification and hybridization to the IBC array (HumanCVD beadchip, Illumina), as previously described [66].

### Plasmid constructs and site-directed mutagenesis

A 2007-bp fragment consisting of the human *LIPG* promoter (1755-bp portion flanking the transcription start site) and the 5′ untranslated region (252-bp) was PCR-amplified from a human *LIPG* plasmid clone with PCR primers that introduced Kpn I and Xho I restriction sites at the 5′ and 3′ ends of the fragment, respectively. This amplified region was cloned into the pGL3-basic vector (Promega) with the Kpn I and Xho I restriction sites to generate a construct with wild-type *LIPG* promoter driving firefly luciferase expression and was confirmed by PCR.

Mutagenesis of the wild-type *LIPG* promoter (firefly luciferase) construct to generate mutant constructs for each of the identified regulatory variants was achieved by using QuikChange Site-Directed Mutagenesis Kit (Stratagene) according to the manufacturer's directions with primer sequences available in Table S3. Plasmids were sequenced after site-directed mutagenesis to confirm the changes and to rule out additional nonspecific changes.

### Cell culture and dual-reporter luciferase assays

Clonetics human umbilical vein endothelial cells (HUVEC, Lonza) were cultured in Clonetics Endothelial Growth Medium (EGM-2, Lonza) at 37°C, 5% (v/v) CO_2_. In preparation for luciferase assays, HUVECs were passaged 3 times and plated (10,000 cells/well) overnight in 96-well tissue culture grade black-and-white microplates (Perkin-Elmer) in EGM-2. Cells were transfected by using 2 µg DNA/well (*LIPG* promoter construct and pRL-SV40 in a 50∶1 ratio) and Fugene HD transfection reagent (Roche) in a 1∶3 ratio of DNA to Fugene HD following the manufacturer's instructions. Cells were harvested at 36 h after transfection. Luciferase assays were performed with the Dual Luciferase Assay Kit (Promega) and a dual-injection microplate luminometer (Orion Microplate Luminometer, Berthold Detection Systems). Each well was normalized by Renilla luciferase luminescence values. Normalized values were compared to wild-type *LIPG* promoter constructs transfected on the same plate. Each construct was transfected with 6 replicate wells for each experiment. Each construct was evaluated at least three times.

### Enzyme-linked immunosorbent assays

The preheparin mass of EL was measured from the plasma of SIRCA study participants genotyped for some of the identified common variants and 2 GWAS-identified noncoding variants. Detailed methods of the EL sandwich ELISA have been reported previously [67], [68]. Briefly, rabbit anti-human EL antibody was used to capture EL from diluted plasma samples, followed by incubation with biotin-conjugated rabbit anti-human EL antibody and streptavidin-horseradish peroxidase conjugate with O-phenylenediamine for detection.

### Statistical analyses

Analysis and comparison of promoter activity between wild-type and variant *LIPG* promoter constructs from the luciferase assays were conducted by using unpaired Student's *t*-tests (*P*-values<0.05 were considered to be statistically significant). Numbers of individuals with a rare variant identified in each sequencing cohort were initially compared using 2-tailed Fisher's exact tests. Variants that did not alter promoter activity *in vitro* were discounted, and individuals harboring these variants were included in their respective sequencing cohort as individuals without a functionally altering variant. Numbers of individuals with variants decreasing promoter activity and with variants decreasing promoter activity in each sequencing cohort were then compared separately using 1-tailed Fisher's exact tests.

The FHS association analysis was completed by performing multiple linear regressions of the residuals of lipid phenotypes, separately by gender, after adjustment for means of age, age^2^, BMI, alcohol intake, and smoking status. In this analysis, for women, the proportion of exams that a woman was menopausal and on hormone replacement therapy was included as a covariate. For association of variant genotypes with effect on plasma EL in SIRCA, plasma EL concentrations were log-transformed to normalize the distribution and analyzed with linear regression. Linkage disequilibrium (LD) calculations and presentation were performed with Haploview software [34].

## Supporting Information

Figure S1 Expression of *LIPG* promoter construct in HUVECs. Relative *LIPG* promoter activity of WT *LIPG* promoter construct (1755-bp of *LIPG* promoter and 5′ UTR driving expression of firefly luciferase) and pGL3-basic construct (no functional promoter) in HUVECs. Firefly luciferase activity of WT construct was normalized to that of cotransfected Renilla luciferase, and Renilla-normalized promoter activity was normalized to that of pGL3-basic construct to determine functionality of WT promoter construct in HUVECs for subsequent analysis of variant constructs. Assays were conducted with 6 replicates per experiment and data is given as mean ± standard deviation. ***P<0.0001 relative to pGL3-basic.
(TIF)

Figure S2 Functional analysis of additional rare *LIPG* regulatory variants identified. Relative promoter activity of rare variants (MAF<0.01) identified from resequencing of individuals with high HDL-C levels (A) or low HDL-C levels (B) which did not alter LIPG promoter activity in vitro, and of rare variants present in both high HDL-C and low HDL-C cohorts (C). Plasmid constructs expressing firefly luciferase under the control of wild-type (WT) or variant *LIPG* promoters were individually co-transfected with a Renilla luciferase reporter construct (pRL-SV40) in HUVECs. Firefly luciferase expression were measured and normalized to that of Renilla luciferase, and Renilla-normalized promoter activities for variant constructs were then normalized to those of the WT construct to provide relative *LIPG* promoter activities of the variants. Assays were conducted with 6 replicates per experiment and data is given as mean ± standard deviation.
(TIF)

Figure S3 Linkage disequilibrium in and surrounding the *LIPG* promoter. LD was estimated for 3 common *LIPG* promoter variants (rs3829632, rs4245232 and rs9958947) and additional common variants upstream and in intronic regions of *LIPG* from HapMap CEU population dataset using Haploview software. Values in the LD plot are estimated squared correlation coefficients (R^2^).
(TIF)

Table S1 Characteristics of participants in sequencing cohorts with rare *LIPG* regulatory variants.
(DOCX)

Table S2 Association of *LIPG* combined haplotype variants with HDL-C in GLGC GWAS.
(DOCX)

Table S3 Primers used for site-directed mutagenesis to generate *LIPG* promoter variant constructs.
(DOCX)
