# Supplementary figures and images for: Mining the LIPG Allelic Spectrum Reveals the Contribution of Rare and Common Regulatory Variants to HDL Cholesterol
Source: PLoS Genet. 2011 Dec 8;7(12):e1002393. doi: 10.1371/journal.pgen.1002393 (PMC3234219; doi:10.1371/journal.pgen.1002393)

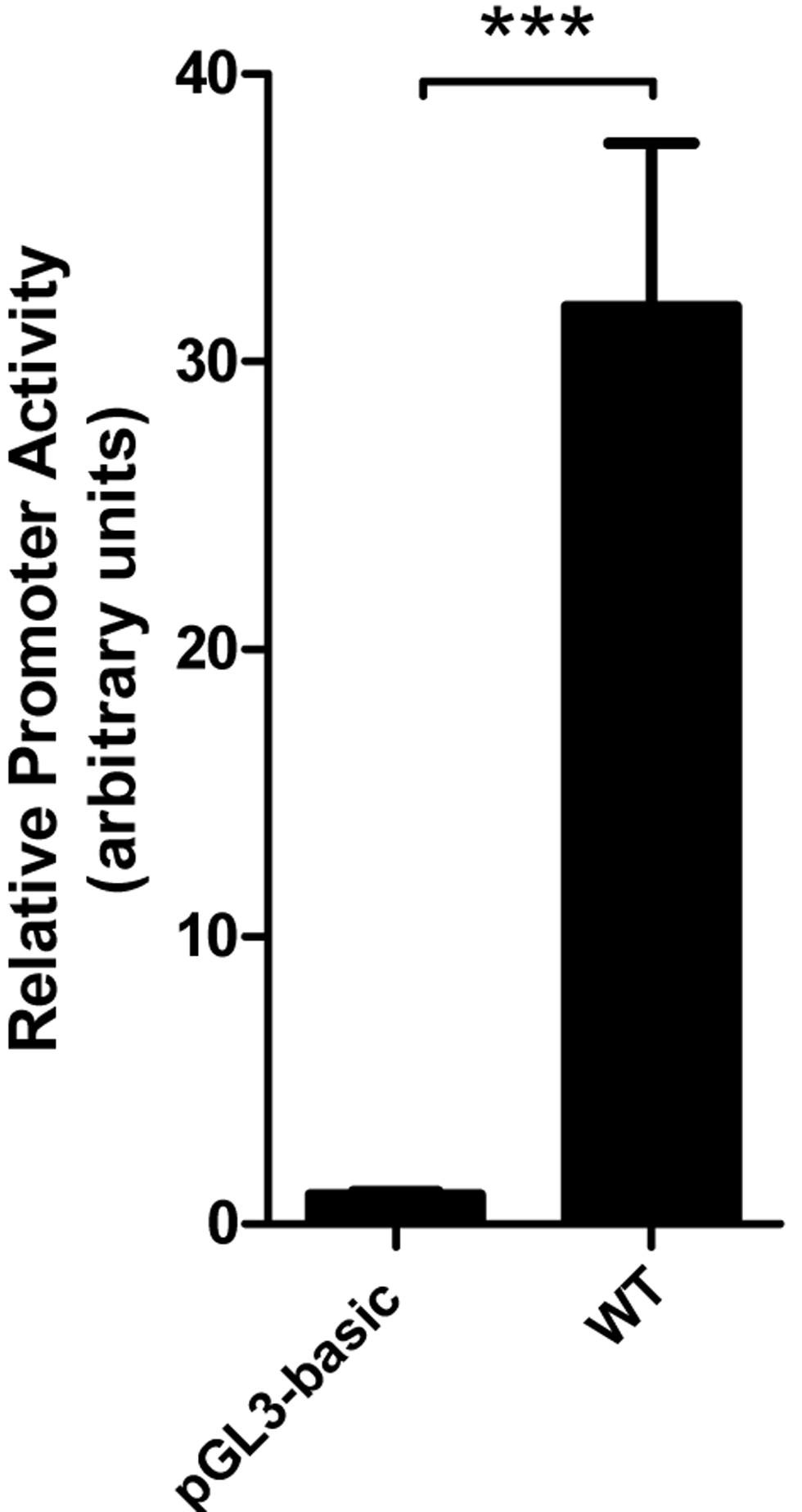

Supplement: Figure S1 — Expression of LIPG promoter construct in HUVECs. Relative LIPG promoter activity of WT LIPG promoter construct (1755-bp of LIPG promoter and 5′ UTR driving expression of firefly luciferase) and pGL3-basic construct (no functional promoter) in HUVECs. Firefly luciferase activity of WT construct was normalized to that of cotransfected Renilla luciferase, and Renilla-normalized promoter activity was normalized to that of pGL3-basic construct to determine functionality of WT promoter construct in HUVECs for subsequent analysis of variant constructs. Assays were conducted with 6 replicates per experiment and data is given as mean ± standard deviation. ***P<0.0001 relative to pGL3-basic. (TIF) [file pgen.1002393.s001.tif]

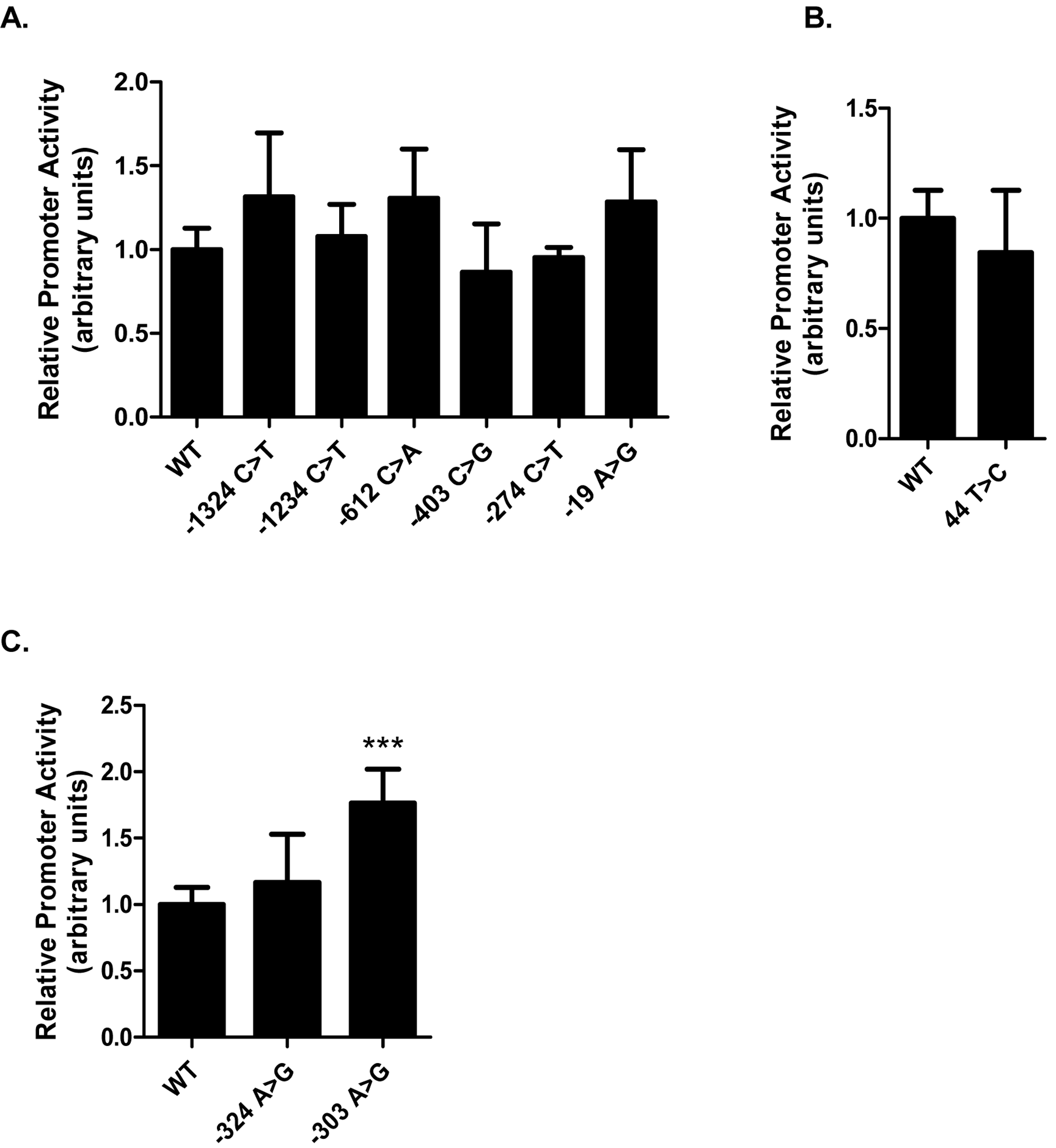

Supplement: Figure S2 — Functional analysis of additional rare LIPG regulatory variants identified. Relative promoter activity of rare variants (MAF<0.01) identified from resequencing of individuals with high HDL-C levels (A) or low HDL-C levels (B) which did not alter LIPG promoter activity in vitro, and of rare variants present in both high HDL-C and low HDL-C cohorts (C). Plasmid constructs expressing firefly luciferase under the control of wild-type (WT) or variant LIPG promoters were individually co-transfected with a Renilla luciferase reporter construct (pRL-SV40) in HUVECs. Firefly luciferase expression were measured and normalized to that of Renilla luciferase, and Renilla-normalized promoter activities for variant constructs were then normalized to those of the WT construct to provide relative LIPG promoter activities of the variants. Assays were conducted with 6 replicates per experiment and data is given as mean ± standard deviation. (TIF) [file pgen.1002393.s002.tif]

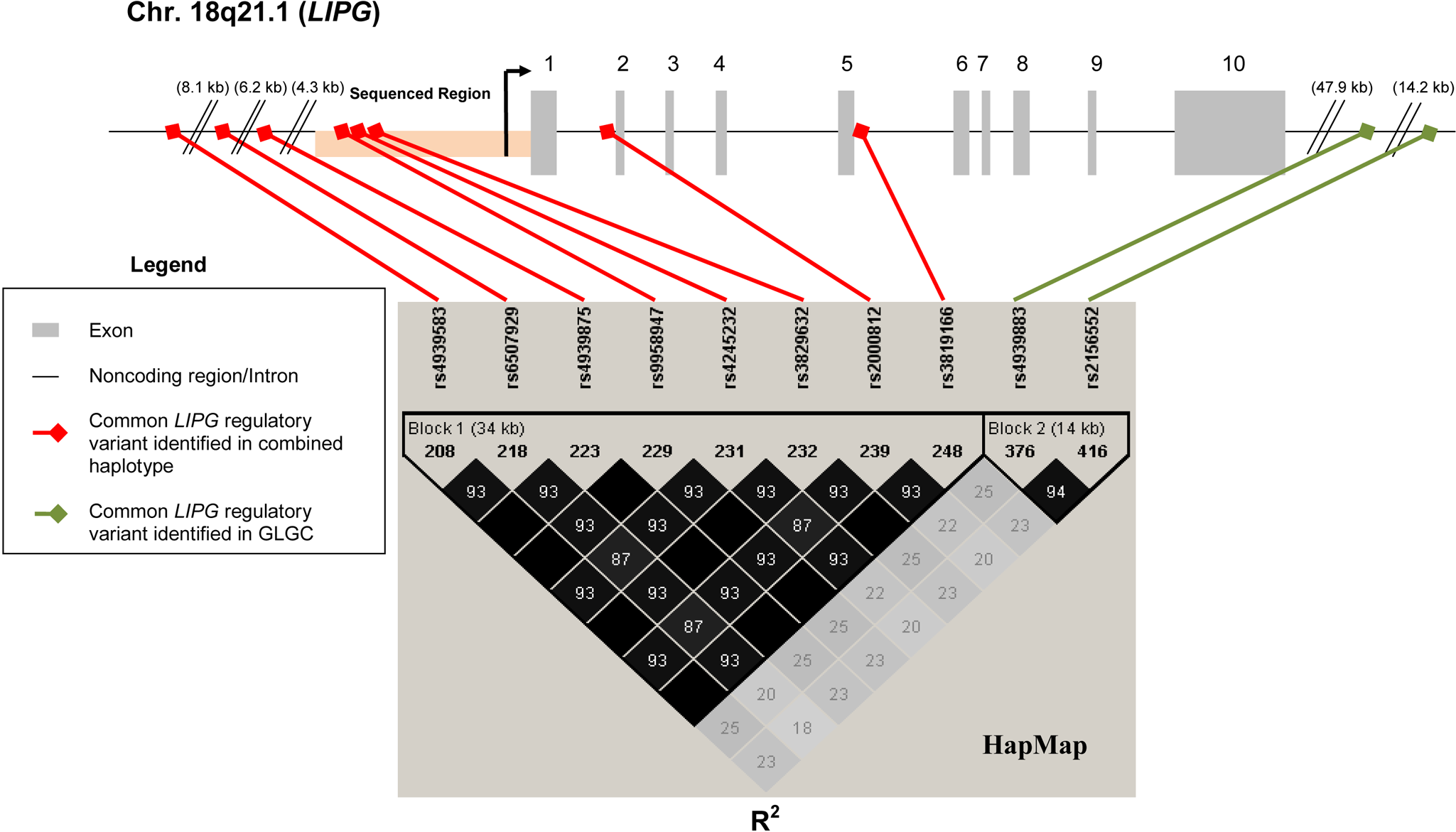

Supplement: Figure S3 — Linkage disequilibrium in and surrounding the LIPG promoter. LD was estimated for 3 common LIPG promoter variants (rs3829632, rs4245232 and rs9958947) and additional common variants upstream and in intronic regions of LIPG from HapMap CEU population dataset using Haploview software. Values in the LD plot are estimated squared correlation coefficients (R2). (TIF) [file pgen.1002393.s003.tif]
