# Supplementary material for: Mining the LIPG Allelic Spectrum Reveals the Contribution of Rare and Common Regulatory Variants to HDL Cholesterol
Source: PLoS Genet. 2011 Dec 8;7(12):e1002393. doi: 10.1371/journal.pgen.1002393 (PMC3234219; doi:10.1371/journal.pgen.1002393)
Supplement: Table S1 — Characteristics of participants in sequencing cohorts with rare LIPG regulatory variants. (DOCX) [file pgen.1002393.s004.docx]

**Table S1: Characteristics of participants in sequencing cohorts with rare *LIPG* regulatory variants**

| **Mutation** | **Participant ID** | **Sequencing Cohort** | **HDL-C (mg/dl)** | **Age (yrs)** | **Gender (M/F)** | **Race** |
| --- | --- | --- | --- | --- | --- | --- |
| -1487 A>G | HHDL0273 | HHDL | 124 | 45 | F | African American |
|  | HHDL0396 | HHDL | 132 | 56 | F | African American |
| -1324 C>T | HHDL1111 | HHDL | 104 | 60 | F | Caucasian |
| -1234 C>T | HHDL0943 | HHDL | 102 | 53 | F | African American |
| -1080 G>T | HHDL0396 | HHDL | 132 | 56 | F | African American |
|  | HHDL0327 | HHDL | 85 | 56 | M | African American |
|  | HHDL0783 | HHDL | 87 | 73 | M | African American |
| -612 C>A | HHDL0435 | HHDL | 152 | 53 | F | Caucasian |
| -537 T>C | HHDL0122 | HHDL | 100 | 51 | F | African American |
| -410 C>G | HHDL0029 | HHDL | 114 | 73 | F | Caucasian |
| -403 C>G | HHDL1024 | HHDL | 102 | 74 | F | Caucasian |
|  | HHDL1217 | HHDL | 108 | 36 | F | Caucasian |
| -274 C>T | HHDL0742 | HHDL | 108 | 41 | F | Caucasian |
|  | HHDL1030 | HHDL | 106 | 84 | F | Caucasian |
| -19 A>G | HHDL0827 | HHDL | 90 | 53 | M | Caucasian |
| -1666 G>C | pcath980099 | LHDL | 36 | 78 | F | Caucasian |
|  | SCOR255 | LHDL | 34 | 40 | M | African American |
| -1223 A>G | pcath981218 | LHDL | 33 | 59 | F | Caucasian |
| -1052 C>A | SCOR286 | LHDL | 34 | 46 | F | Caucasian |
| -175 G>A | hcath0235 | LHDL | 37 | 76 | F | Caucasian |
| 44 T>C | HHDL1141 | LHDL | 38 | 74 | F | Caucasian |
| -324 A>G | HHDL0943 | HHDL | 102 | 53 | F | African American |
|  | HHDL0803 | HHDL | 85 | 64 | M | African American |
|  | HHDL1505 | HHDL | 82 | 66 | M | Caucasian |
|  | PAM0218 | LHDL | 44 | 38 | F | African American |
| -303 A>G | HHDL0273 | HHDL | 124 | 45 | F | African American |
|  | PAM0183 | LHDL | 36 | 34 | F | African American |
|  | PAM0205 | LHDL | 28 | 51 | M | African American |
