# Supplementary material for: Mining the LIPG Allelic Spectrum Reveals the Contribution of Rare and Common Regulatory Variants to HDL Cholesterol
Source: PLoS Genet. 2011 Dec 8;7(12):e1002393. doi: 10.1371/journal.pgen.1002393 (PMC3234219; doi:10.1371/journal.pgen.1002393)
Supplement: Table S2 — Association of LIPG combined haplotype variants with HDL-C in GLGC GWAS. (DOCX) [file pgen.1002393.s005.docx]

**Table S2: Association of *LIPG* combined haplotype variants with HDL-C in GLGC GWAS**

| **Variant** | **Position on Chr.18**^a^ | **Effect of Minor Allele on HDL-C (P value)**^b^ | **Number of Subjects** ^c^ |
| --- | --- | --- | --- |
| rs4939583 | 45321944 (upstream) | ↓ (4.53×10^-7^) | 98392 |
| rs6507929 | 45330061 (upstream) | ↓ (4.15×10^-8^) | 98409 |
| rs4939875 | 45347788 (upstream) | ↓ (1.60×10^-8^) | 98408 |
| rs9959847 (-1495 T>C) | 45340930 (promoter) | ↓ (7.71×10^-9^) | 98409 |
| rs4245232 (-1429 C>A) | 45340996 (promoter) | ↓ (8.64×10^-10^) | 98409 |
| rs3829632 (-1309 A>G) | 45341116 (promoter) | ↓ (5.31×10^-5^) | 36612 |
| rs2000812 | 45347788 (intron 2) | ↓ (2.52×10^-9^) | 98409 |
| rs3819166 | 45356100 (intron 5) | ↓ (3.77×10^-9^) | 98409 |

^a^Chromosomal position from HapMap Project data (<http://hapmap.ncbi.nlm.nih.gov/cgi-perl/gbrowse/hapmap28_B36/>).

^b^Association with HDL-C in Global Lipids Genetics Consortium GWAS (13).

^c^From GLGC GWAS.
