# Supplementary material for: Mining the LIPG Allelic Spectrum Reveals the Contribution of Rare and Common Regulatory Variants to HDL Cholesterol
Source: PLoS Genet. 2011 Dec 8;7(12):e1002393. doi: 10.1371/journal.pgen.1002393 (PMC3234219; doi:10.1371/journal.pgen.1002393)
Supplement: Table S3 — Primers used for site-directed mutagenesis to generate LIPG promoter variant constructs. (DOCX) [file pgen.1002393.s006.docx]

**Table S3: Primers used for site-directed mutagenesis to generate *LIPG* promoter variant constructs**

| **Mutation**^a^ | **Primer Direction** | **Site-Directed Mutagenesis Primer**^b^ |
| --- | --- | --- |
| -1666 G>C | Forward | GCTCACATTTAAAGGTCAATTG***C***CCAAATTGAACCTAGAGCAG |
|  | Reverse | CTGCTCTAGGTTCAATTTGG***G***CAATTGACCTTTAAATGTGAGC |
| -1495 T>C | Forward | CAGAGTGGGAACAACATATC***C***GGGGTGATATTTACC |
|  | Reverse | GGTAAATATCACCCC***G***GATATGTTGTTCCCACTCTG |
| -1487 A>G | Forward | GGGAACAACATATCTGGGGGTG***G***TATTTACCATCGC |
|  | Reverse | GCGATGGTAAATA***C***CACCCCCAGATATGTTGTTCCC |
| -1429 C>A | Forward | CTAAAGCCCGCCTG***A***GTCTTGTTAATGAATGATAGCACAC |
|  | Reverse | GTGTGCTATCATTCATTAACAAGAC***T***CAGGCGGGCTTTAG |
| -1358 T insertion | Forward | CAGCTGGAGTAACAAAAG***T***TAATGAGGCGAGGTCGG |
|  | Reverse | CCGACCTCGCCTCATTA***A***CTTTTGTTACTCCAGCTG |
| -1324 C>T | Forward | CAGGATCTGCTGGCTC***T***GGCTCGGTTTCCTC |
|  | Reverse | GAGGAAACCGAGCC***A***GAGCCAGCAGATCCTG |
| -1309 G>A | Forward | GCTCGGTTTCCTC***G***CACAGGTCTCTCCAG |
|  | Reverse | CTGGAGAGACCTGTG***C***GAGGAAACCGAGC |
| -1234 C>T | Forward | GGTTTTCCCTCCC***T***AGGCAGGGGCAGGAC |
|  | Reverse | GTCCTGCCCCTGCCT***A***GGGAGGGAAAACC |
| -1223 A>G | Forward | GGCAGGGGC***G***GGACCTGTTCCGGGCG |
|  | Reverse | CGCCCGGAACAGGTCC***C***GCCCCTGCC |
| -1080 G>T | Forward | GTTGTTGGCGCAGCC***T***CCCCGTCGGTG |
|  | Reverse | CACCGACGGGG***A***GGCTGCGCCAACAAC |
| -1052 C>A | Forward | GGGCTCAGTCCC***A***GCTCCCCAGCGCC |
|  | Reverse | GGCGCTGGGGAGC***T***GGGACTGAGCCC |
| -612 C>A | Forward | CTAGTGGCGGAG***A***GAGCATTCCGAGCAGG |
|  | Reverse | CCTGCTCGGAATGCTC***T***CTCCGCCACTAG |
| -537 T>C | Forward | CCGCCGGGTTA***C***TGTGCGGCCGCGC |
|  | Reverse | GCGCGGCCGCACA***G***TAACCCGGCGG |
| -410 C>G | Forward | GGGAGCGGATAGA***G***CACTCCCAGAGAG |
|  | Reverse | CTCTCTGGGAGTG***C***TCTATCCGCTCCC |
| -403 C>G | Forward | GCGGATAGACCACTCC***G***AGAGAGAGTGTGG |
|  | Reverse | CCACACTCTCTCT***C***GGAGTGGTCTATCCGC |
| -324 A>G | Forward | GACGCAGAGTTTC***G***GGGAAATGTCCGCCTC |
|  | Reverse | GAGGCGGACATTTCCC***C***GAAACTCTGCGTC |
| -303 A>C | Forward | GTCCGCCTCCGCC***C***CTTGGGATGGCAG |
|  | Reverse | CTGCCATCCCAAG***G***GGCGGAGGCGGAC |
| -274 C>T | Forward | GGGAGAGGAGGAT***T***TGGGTGTCCGGAGG |
|  | Reverse | CCTCCGGACACCCA***A***ATCCTCCTCTCCC |
| -175 G>A | Forward | GAGCGTGCGGC***A***TCCACGCGGTGAGTGTG |
|  | Reverse | CACACTCACCGCGTGGA***T***GCCGCACGCTC |
| -19 A>G | Forward | CCCATACTTTTAAAAACT***G***CCTCTATAGGAGCGTGACAGC |
|  | Reverse | GCTGTCACGCTCCTATAGAGG***C***AGTTTTTAAAAGTATGGG |
| 44 5' UTR T>C | Forward | CAGGACGAGGGCAGA***C***CTCGTTCTGGGGC |
|  | Reverse | GCCCCAGAACGAG***G***TCTGCCCTCGTCCTG |
| 229 5' UTR T>G | Forward | CTTCTGTTTCT***G***GGGAGGGGGTGTGGCG |
|  | Reverse | CGCCACACCCCCTCCC***C***AGAAACAGAAG |

^a^Relative to transcription start site. ^b^Nucleotide change relative to WT sequence shown in bold and underlined.
